# The relationship of soluble neuropilin-1 to severe COVID-19 risk factors in polycystic ovary syndrome

**DOI:** 10.1016/j.metop.2021.100079

**Published:** 2021-01-18

**Authors:** Abu Saleh Md Moin, Thozhukat Sathyapalan, Stephen L. Atkin, Alexandra E. Butler

**Affiliations:** Diabetes Research Center (DRC), Qatar Biomedical Research Institute (QBRI), Hamad Bin Khalifa University (HBKU), Qatar Foundation (QF), 34110, Doha, Qatar; Academic Endocrinology, Diabetes and Metabolism, Hull York Medical School, Hull, UK; Royal College of Surgeons in Ireland Bahrain, Adliya, Bahrain; Diabetes Research Center (DRC), Qatar Biomedical Research Institute (QBRI), Hamad Bin Khalifa University (HBKU), Qatar Foundation (QF), PO Box 34110, Doha, Qatar

**Keywords:** Neuropilin-1, Polycystic ovary syndrome, COVID-19, SARS-CoV-2

**To the editor**

The SARS-CoV-2 coronavirus enters target cells via the angiotensin-converting enzyme 2 (ACE2) receptor; however, ACE2 expression does not match SARS-CoV-2 tissue load, suggesting additional co-factors are required for viral entry [1]. Neuropilin-1 (NRP1) is such a co-factor that, when expressed alone shows minimal viral infectivity, but when co-expressed with ACE2 markedly increases viral infectivity [1]. NRP1 is a transmembrane glycoprotein, which is expressed in endothelial cells, and serves as a receptor for vascular endothelial growth factor (VEGF) [2], and both NRP1 and VEGF expression are increased in COVID-19 patients [3,4]. SARS-CoV-2 uses the viral spike protein for cell entry, cleaving the protein that then attaches to NRP1 [5]. Therefore, tissues enriched for NRP1 have increased infectivity risk [1] and subjects expressing increased NRP1 may have increased risk.

The Renin-Angiotensin System (RAS) system regulates blood pressure; its overactivity is reported in metabolic syndrome, obesity and type 2 diabetes (T2D) [6], all high-risk conditions for severe COVID-19 disease. Polycystic ovary syndrome (PCOS), a cardiometabolic disease encompassing metabolic syndrome, insulin resistance [7] and obesity with increased T2D risk [8], also predisposes to severe COVID-19 disease, as suggested by case reports of PCOS women infected by SARS-CoV-2 [9]. We have reported higher renin and lower angiotensinogen levels, indicating RAS hyperactivation, in PCOS [10]. Since RAS-related proteins, NRP1 and VEGF, are implicated in COVID-19 severity, we hypothesized that increased risk of severe COVID-19 in PCOS may be reflected in altered soluble NRP1 (sNRP1) levels and their association with RAS-related proteins and VEGF.

“146 PCOS and 97 control women who presented sequentially to the Department of Endocrinology, Hull and East Yorkshire Hospitals NHS Trust were recruited to the local PCOS biobank (ISRCTN70196169)” [10]. “PCOS diagnosis was based on all three Rotterdam consensus diagnostic criteria, these specifically being hyperandrogenism, either clinical or biochemical with a Ferriman-Gallwey score of >8 and free androgen index >4.5, oligomenorrhea (defined as ≤9 menses/year) or amenorrhea (defined as lack of menses for ≥3-months) and polycystic ovaries (≥12 antral follicles in ≥1 ovary or ovarian volume of ≥10cm^3^) as determined by transvaginal ultrasonography [11]. All participants were determined to have no concurrent illness, were not taking any medication for the prior 9-months and had no plans to conceive. All women in the PCOS cohort also fulfilled NIH criteria for PCOS diagnosis.

All women underwent the following biochemical testing: prolactin, 17beta hydroxyprogesterone, thyroid function tests (TFTs), and dehydroepiandrosterone sulfate (DHEAS) in order to ensure exclusion of other confounders. Body mass index (BMI), waist and hip circumference, height and weight were documented. Control women had normal regular menses and androgen levels within normal limits, thereby excluding PCOS by both NIH and Rotterdam criteria. The trial was conducted in accordance with the International Conference on Harmonization Good Clinical Practice (ICHGCP) and the Declaration of Helsinki [12].

“After collection, blood samples were measured in the Chemistry Laboratory, Hull Royal Infirmary, UK. Using an immunometric assay with fluorescence detection on the DPC Immulite 2000 analyzer (Euro/DPC, Llanberis, UK) and following the manufacturer’s recommended protocol, TSH, prolactin, insulin, C reactive protein (CRP), DHEAS, and sex hormone binding globulin (SHBG) levels were determined [13]. Using isotope dilution liquid chromatography-tandem mass spectrometry, testosterone levels were determined (Waters Corporation, Manchester, UK) in the UK, as previously described [14].

The free androgen index (FAI) was calculated as follows: total testosterone x 100/SHBG. For insulin, the analytical sensitivity of the assay was 2μU/ml, with coefficient of variation (COV) 6%, and no stated cross-reactivity with proinsulin. Plasma glucose was measured with a Synchron LX 20 analyzer (Beckman-Coulter), and again using the manufacturer’s recommended protocol, with COV for the assay 1.2% at a mean glucose concentration of 5.3mmol/L during the study. Insulin resistance was calculated using the HOMA method as follows: [HOMA-IR= (insulin x glucose)/22.5]” [13]. All analyses were performed following current guidelines, regulations and quality control” [12].

Circulating NRP1 (sNRP1) levels, RAS-related proteins (ACE2, Renin, Angiotensinogen (AGT)) and VEGF were determined by Slow Off-rate Modified Aptamer (SOMA)-scan plasma protein measurement [15]. “To quantify protein levels, the SOMAscan assay was performed using the Tecan Freedom EVO liquid handling system (Tecan Group, Maennedorf, Switzerland); buffers and SOMAmers were provided with the SOMAscan HTS Assay 1.3K plasma kit (SomaLogic, Boulder, CO). Assays were performed according to manufacturer’s instructions as described previously [16,17] utilizing 96-well plates, each containing a maximum of 85 plasma samples, plus 3 ‘quality control’ and 5 ‘calibrator’ plasma samples. In brief, EDTA plasma samples were diluted (bins of 40%, 1% and 0.05%), then incubated with streptavidin-coated beads that were immobilized with dilution-specific SOMAmers using a photocleavable linker and biotin. After a washing step, bound proteins were biotinylated then released from the beads by photocleavage of the SOMAmer-bead linker. The liberated SOMAmer-protein complexes were then treated with a polyanionic competitor to disrupt non-specific binding and recaptured on a second set of streptavidin-coated beads. After thorough washing, 5′ Cy3 fluorophore-labelled SOMAmers were released under denaturing conditions, hybridized on microarray chips with SOMAmer-complementary sequences and scanned using a SureScan G2565 Microarray Scanner (Agilent, Santa Clara, CA).

Initial Relative Fluorescent Units (RFUs) were obtained from microarray intensity images using Agilent Feature Extraction Software (Agilent, Santa Clara, CA). Raw RFUs were normalized and calibrated using software provided by SomaLogic. This included (a) microarray hybridization normalization based on spiked-in hybridization controls, (b) plate-specific intensity normalization, (c) median signal normalization, and (d) median calibrator scaling of single RFU intensities according to calibrator reference values. Samples with a high degree of hemolysis (Haptoglobin log RFU<10) were excluded from the analysis.

Statistical analyses were performed on log_2_ RFU values using R version 3.5.2 (R Foundation for Statistical Computing, Vienna, Austria) including base R package. Data handling and differential protein expression were analyzed using the autonomics and limma [18] packages. For differential protein analysis, limma models containing contrasts between PCOS and control subjects were applied. Blocking by patient ID was undertaken to account for random effects. Batch effect correction was performed by adding batch as a covariate to the model. Limma obtained p-values were corrected using the Benjamini-Hochberg method [19].

There are no studies detailing the changes in neuropilin protein in PCOS versus control women on which to base a power calculation. Sample size requirements for pilot studies was reviewed by Birkett and Day [20] wherein they concluded that, to estimate effect size and variability, a minimum of 20 degrees-of-freedom is necessary. Hence, here, analysis of samples from 20 patients minimum per group was required. Data trends were evaluated, with non-parametric tests applied to data that violated the assumptions of normality when tested using the Kolmogorov-Smirnov Test. Comparison between groups was performed using Student’s t-test where a p-value <0.05 was taken as significant. Statistical analysis was performed using Graphpad Prism (San Diego, CA, USA)” [21].

As reported previously [10], cohorts were age-matched, but PCOS women had increased insulin resistance, androgens and CRP (p < 0.001); systolic and diastolic blood pressure, and waist circumference were higher (p < 0.05). As reported, RAS system overactivity was found with elevated renin, and decreased angiotensinogen and ACE2 in PCOS (p < 0.05), indicative of increased hypertensive risk [10].

sNRP1 was decreased in PCOS (2117 ± 40 vs 2287 ± 52 RFU, PCOS vs control; p = 0.008); VEGF levels did not differ (8831.7 ± 97.5 vs 8708.5 ± 8708.5 RFU VEGF, PCOS vs control, p = ns). sNRP1 did not correlate with VEGF, ACE2 or renin; a negative correlation with angiotensinogen was seen for PCOS (r = −0.26; p = 0.0045) that trended in controls (r = 0.2; p = 0.06) (Fig. 1).

**Fig. 1:**
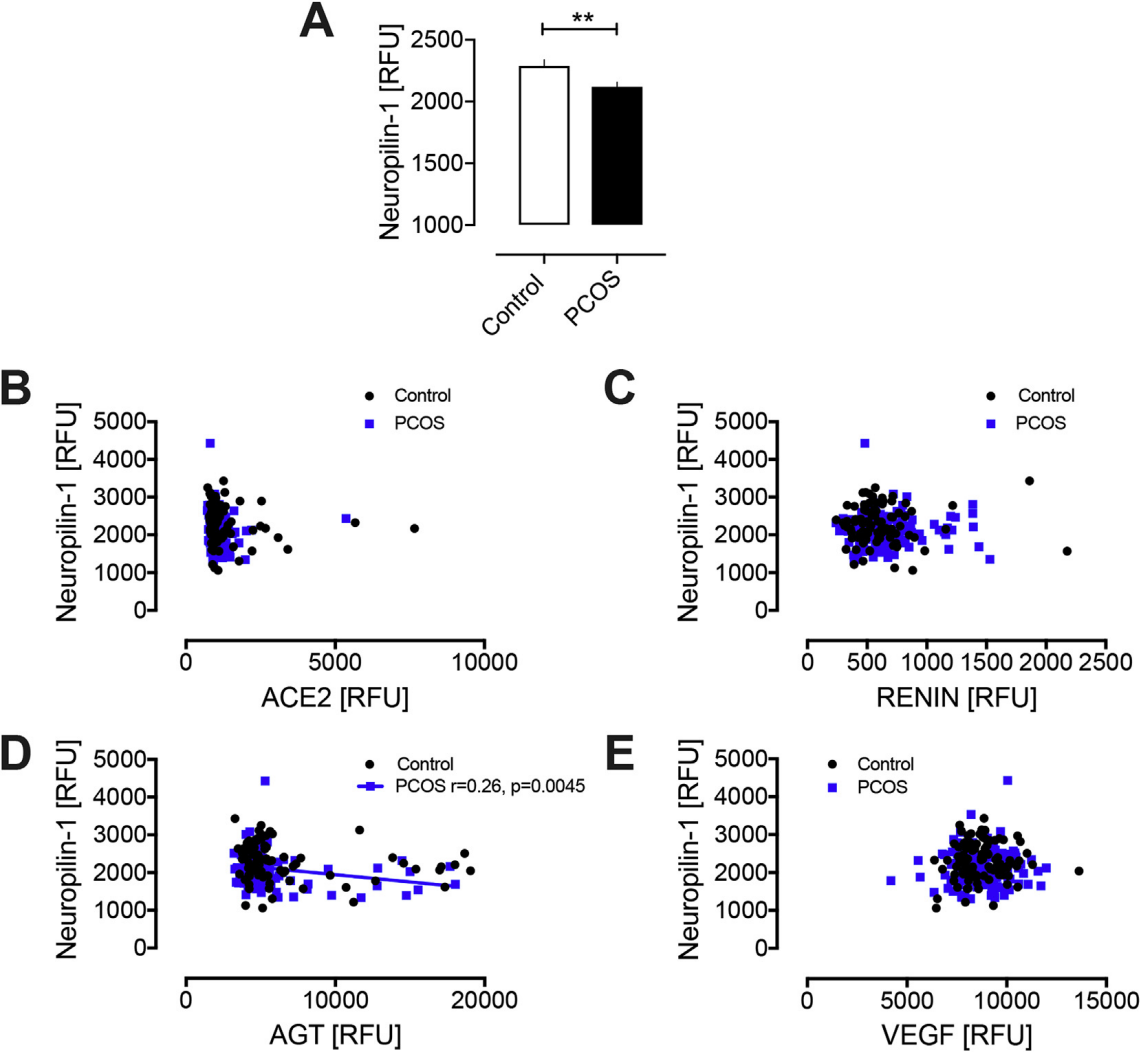
Reduction in neuropilin-1 in PCOS women in comparison to control subjects (A); relationship of neuropilin-1 with ACE2 (B), Renin (C) Angiotensinogen (D) and VEGF (E) in PCOS and control women. AGT, angiotensinogen; VEGF, vascular endothelial growth factor; RFU, relative fluorescent units; ∗∗p < 0.01.

Lower levels of sNRP1in PCOS may reflect reduced ‘ligand-trapping’ of VEGF in PCOS. sNRP1 isoforms may regulate NRP1 activity [22,23]; therefore, no change in VEGF levels with lower sNRP1 would reflect in higher endogenous membrane-bound NRP1 in PCOS, resulting in higher SARS-CoV-2 infectivity. Moreover, sNRP1 negatively correlated with RAS activation through angiotensinogen, and higher RAS activation may increase COVID-19 disease risk.

The strengths of this study were the relatively large, age matched and ethnically homogeneous cohorts, in addition to the use of state-of-the-art laboratory platforms for biochemical analysis, including gold standard tandem mass spectroscopy for measurement of testosterone. A limitation is that the women attended a secondary care facility, and this may have introduced an element of bias relative to the general population. Also, because the subjects were all Caucasian, the findings may not be generalizable to other ethnic populations.

In conclusion, sNRP1 levels were lower in PCOS women and correlated with increased RAS activation, suggesting that lower plasma sNRP1 levels may indicate increased COVID-19 disease susceptibility.

## Declarations

*Ethics approval and consent to participate:* The Newcastle & North Tyneside Ethics committee approved this study that was conducted according to the Declaration of Helsinki. All study participants signed an informed consent form prior to participation.

## Consent for publication

All authors gave their consent for publication.

## Availability of data and materials

All the data for this study will be made available upon reasonable request to the corresponding author.

## Funding

No funding was received to perform this study.

## Author contributions

ASMM and AEB analyzed the data and wrote the manuscript. TS supervised clinical studies and edited the manuscript. SLA contributed to study design, data interpretation and the writing of the manuscript. All authors reviewed and approved the final version of the manuscript. Alexandra E Butler is the guarantor of this work.

## Declaration of competing interest

No authors have any conflict of interest or competing interests to declare.
